# Mutational screens highlight glycosylation as a modulator of colony-stimulating factor 3 receptor (CSF3R) activity

**DOI:** 10.1016/j.jbc.2023.104755

**Published:** 2023-04-26

**Authors:** Michael J. Hollander, Stacy A. Malaker, Nicholas M. Riley, Idalia Perez, Nayla M. Abney, Melissa A. Gray, Julia E. Maxson, Jennifer R. Cochran, Carolyn R. Bertozzi

**Affiliations:** 1 Department of Bioengineering, Stanford University, Stanford, California, USA; 2 Department of Chemistry and Sarafan ChEM-H, Stanford University, Stanford, California, USA; 3 Knight Cancer Institute, Oregon Health & Science University, Portland, Oregon, USA; 4 Department of Chemical Engineering, Stanford University, Stanford, California, USA; 5 Howard Hughes Medical Institute, Stanford, California, USA

**Keywords:** glycoprotein, receptor, neutrophil, mutagenesis, mass spectrometry (MS)

## Abstract

The colony-stimulating factor 3 receptor (CSF3R) controls the growth of neutrophils, the most abundant type of white blood cell. In healthy neutrophils, signaling is dependent on CSF3R binding to its ligand, CSF3. A single amino acid mutation in CSF3R, T618I, instead allows for constitutive, ligand-independent cell growth and leads to a rare type of cancer called chronic neutrophilic leukemia. However, the disease mechanism is not well understood. Here, we investigated why this threonine to isoleucine substitution is the predominant mutation in chronic neutrophilic leukemia and how it leads to uncontrolled neutrophil growth. Using protein domain mapping, we demonstrated that the single CSF3R domain containing residue 618 is sufficient for ligand-independent activity. We then applied an unbiased mutational screening strategy focused on this domain and found that activating mutations are enriched at sites normally occupied by asparagine, threonine, and serine residues—the three amino acids which are commonly glycosylated. We confirmed glycosylation at multiple CSF3R residues by mass spectrometry, including the presence of GalNAc and Gal-GalNAc glycans at WT threonine 618. Using the same approach applied to other cell surface receptors, we identified an activating mutation, S489F, in the interleukin-31 receptor alpha chain. Combined, these results suggest a role for glycosylated hotspot residues in regulating receptor signaling, mutation of which can lead to ligand-independent, uncontrolled activity and human disease.

Cell surface receptors direct the first step of signaling pathways that govern many cellular activities (1). Activation of receptor signaling commonly occurs when a ligand binds to the receptor. This binding event often changes the conformation or oligomerization of the receptor, thereby transmitting the signal across the cell membrane (2). As a result, signaling proceeds in a controlled manner only in the presence of the ligand, allowing cells to maintain homeostasis. Examples exist of amino acid mutations which enable receptors to signal even in the absence of ligand, overriding this control mechanism (3). This is especially notable for activating mutations in growth factor receptors which cause cells to proliferate uncontrollably, leading to several forms of cancer (4).

Beyond amino acid substitutions, there is a need to consider the implications for altered posttranslational modifications in dysregulated growth factor receptor signaling. A predominant example is glycosylation, which typically modifies asparagine, serine, or threonine residues with complex glycans. N-linked glycosylation is named for sugar moieties attached to the nitrogen of asparagine in an Asn-X-Ser/Thr sequon, while O-linked glycosylation acts on the oxygen of serine or threonine (5). Glycans are much larger than amino acid side chains and can introduce negative charge through specific monosaccharides such as sialic acids or through sulfate modifications (6). As a result, when glycosylation sites are either introduced or removed by mutation, the effects can potentially be more consequential to overall protein structure than simple amino acid side chain substitutions.

A major challenge of studying protein glycosylation is the difficulty in assigning the sites and structures of the attached glycans. Glycosylation is not genetically templated, so while it is possible for any threonine or serine to be glycosylated, the modification depends on the presence of enzymes, predominantly glycosyltransferases, that build glycans at specific amino acid residues (7). Similarly, asparagine residues that are part of the defined glycosylation sequon may or may not be glycosylated depending on these enzymes and other factors (8). When residues are glycosylated, there is additional macroheterogeneity—the fraction of sites that are occupied—and microheterogeneity—the range of glycan structures at individual sites (9). Computational tools that predict glycosylation based on the sequence of a protein are still in development and require experimental validation (10). We therefore may suspect that a threonine, serine, or asparagine residue is glycosylated but cannot have certainty without direct molecular characterization, typically by mass spectrometry (MS)-based glycoproteomic analysis (11).

Colony-stimulating factor 3 receptor (CSF3R, also G-CSFR) is an example of a cell surface growth factor receptor whose signaling activity was recently proposed to be regulated by glycosylation (12). WT CSF3R is a transmembrane protein that dimerizes in the presence of the ligand colony-stimulating factor 3 (CSF3) with a 2:2 ligand-receptor stoichiometry, leading to downstream JAK/STAT signaling and the development and proliferation of neutrophils, often in the context of infection where an innate immune response is beneficial (13, 14). CSF3R is comprised of an Ig-like domain at the N-terminus for ligand binding, five extracellular fibronectin-III (FnIII) domains, a transmembrane region, and an intracellular signaling domain (Fig. 1*A*) (15). Maxson *et al.* discovered that a cancer driving mutation, T618I in the fifth FnIII (5FnIII) domain, is present in over 80% of cases of chronic neutrophilic leukemia (CNL), a myeloproliferative neoplasm characterized by high levels of mature neutrophils and band cells (16, 17). CNL is distinguished from other related malignancies by the presence of a CSF3R mutation and the absence of other genetic markers, such as the Philadelphia chromosome. The average survival for patients with CNL is approximately two years after diagnosis (18). Treatment options include hydroxyurea, bone marrow transplant, and the small molecule JAK inhibitor, ruxolitinib, which targets signaling downstream of CSF3R (19).

**Figure 1:**
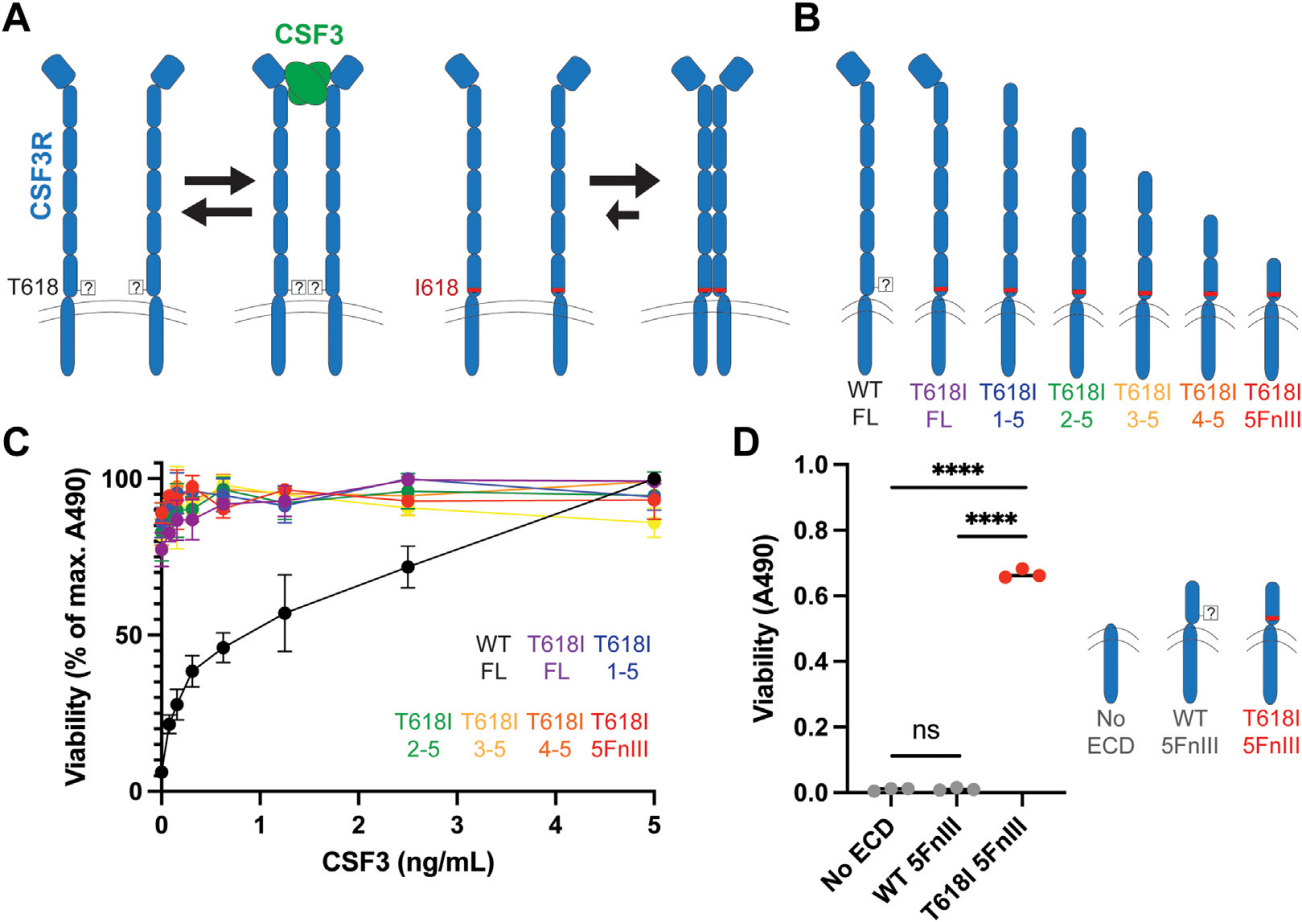
**The membrane-proximal domain initiates ligand-independent signaling.***A*, model for increased ligand-independent dimerization and signaling with the T618I mutation if it removes steric hindrance caused by a glycan at that site. *B*, full-length and truncated constructs of CSF3R for determining which domain(s) are sufficient for activity. *C*, dose-response curves for viability as measured by MTS assay. Values represent mean ± SD. *D*, comparison of mutant and WT fifth FnIII domains and a control without the extracellular domain. Statistical significance was determined by unpaired *t* test (∗∗∗∗*p* < 0.000001, ns: *p* = 0.64). Experiments were conducted at least two times with consistent results. CSF3R, colony-stimulating factor 3 receptor; MTS, 3-(4,5-dimethylthiazol-2-yl)-5-(3-carboxymethoxyphenyl)-2-(4-sulfophenyl)-2H-tetrazolium; FnIII, fibronectin-III.

Maxson *et al.* demonstrated ligand-independent activity of CSF3R with the T618I mutation *in vitro* and showed that it recapitulates a CNL-like disease in a mouse model (20). Furthermore, cells expressing the T618I mutant CSF3R proliferate even in the absence of CSF3 or other growth factor support. It was hypothesized that one key difference between WT threonine and the isoleucine substitution is that threonine can be modified by O-linked glycosylation, while isoleucine cannot; however, direct analysis of this putative glycosylation modification has not yet been reported. A more recently discovered leukemia-associated CSF3R mutation, N610H, does lead to a loss of N-linked glycosylation, as we recently determined using MS methods (21).

In this report, we performed an in-depth analysis of WT CSF3R to determine the effects of glycosylation on receptor signaling and cell growth. We identified the mutant membrane-proximal FnIII domain as the sole initiator of receptor signaling activity in the absence of the ligand CSF3. In addition, we performed a mutational screen for variants that induce CSF3R activation and found that a preponderance of activating mutations were ones that disrupted potential glycosylation sites. Finally, we expanded the screens to related receptors to discover whether these mechanisms are conserved beyond CSF3R. This study points to glycosylation of juxtamembrane domains of cell surface receptors as a regulatory modification that serves to curb aberrant activity.

## Results

### The fifth FnIII domain of CSF3R is sufficient for cell viability

To probe how the T618I mutation controls CSF3R activity, we sought to determine if the mutation has a local effect within the 5FnIII domain or global effects on the complete CSF3R conformation. A working model is that glycosylation could add steric hindrance at residue 618, whereas its removal by mutation could allow increased access for ligand-independent dimerization and signaling (Fig. 1*A*). We expressed individual CSF3R domain fragments on the surface of mammalian cells to determine the minimal receptor regions required for activity. Mouse pro-B BaF3 cells are a model system for studying ligand-independent activity and have been applied to validate clinical mutations and screen for additional variants in mechanistic and biochemical studies (22, 23). Unlike most cultured cells which require only a base medium and serum for growth, BaF3 cells need either interleukin-3 (IL-3) supplementation to signal through endogenous IL-3 receptor or an orthogonal signaling axis (24). In this way, cells containing a signaling activating mutation have a growth advantage in media without IL-3.

We compared BaF3 activity for full-length WT and T618I CSF3R constructs to T618I variants with an increasing number of domains truncated from the N-terminus (Fig. 1*B*). We observed the expected ligand-dependent response for full-length WT CSF3R and ligand-independent viability for cells expressing full-length T618I CSF3R. Interestingly, the 5FnIII T618I domain alone was sufficient for ligand-independent proliferation in a 3-(4,5-dimethylthiazol-2-yl)-5-(3-carboxymethoxyphenyl)-2-(4-sulfophenyl)-2H-tetrazolium (MTS)-based viability assay (Fig. 1*C*). The T618I mutation is critical, as the WT sequence of the 5FnIII construct does not confer activity. To determine whether intermolecular interactions stem from the 5FnIII or intracellular domain for T618I, we expressed a control containing only the transmembrane alpha helix and intracellular domain (residues 621–836) (Fig. 1*D*). This control construct also did not yield viability, suggesting that interactions occur in the 5FnIII domain and that the WT threonine is not permissive to these contacts. We therefore focused on the 5FnIII domain as the region of interest for mutational screens.

### Hydrophobic amino acids are enriched at residue 618

**Figure 2:**
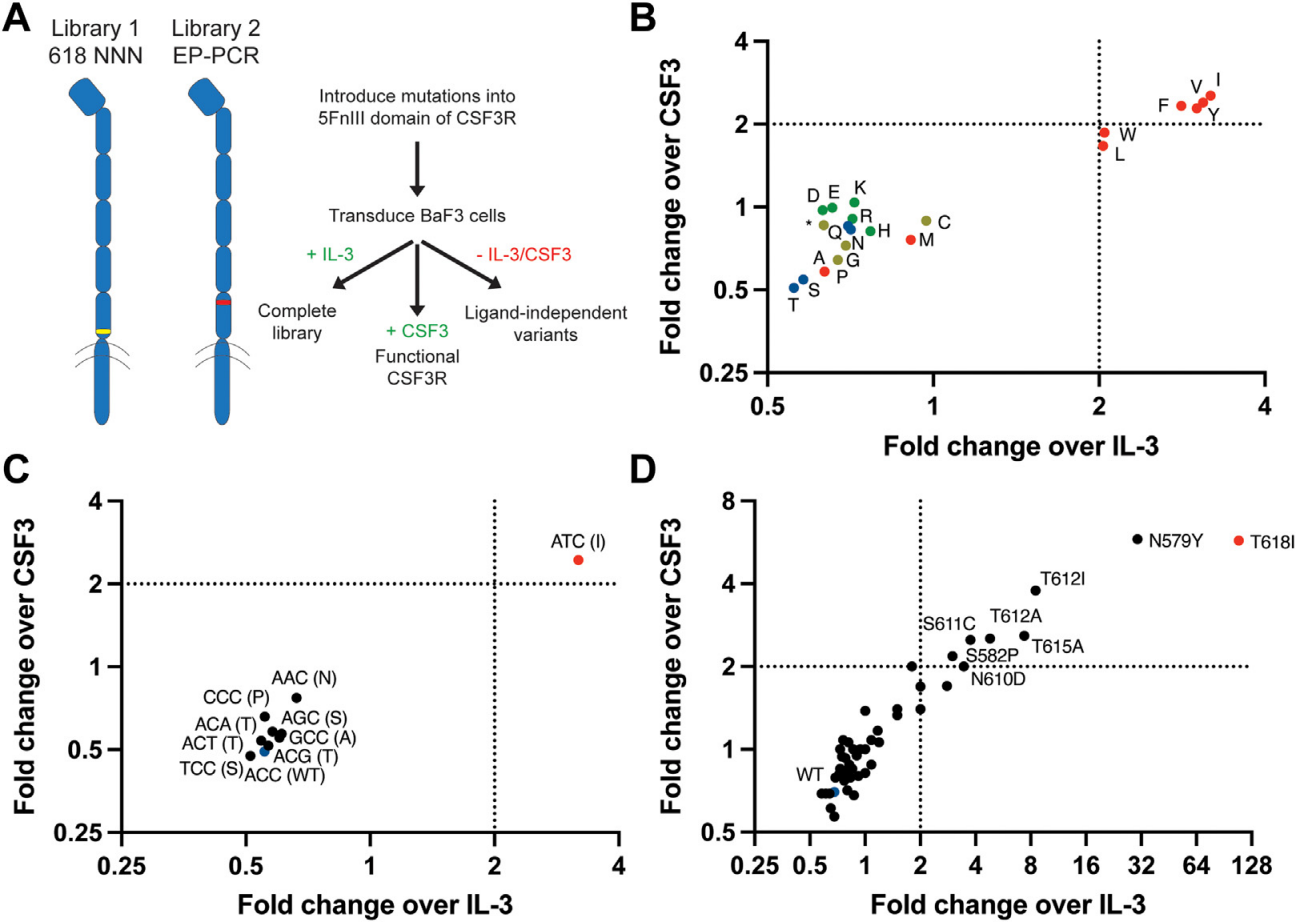
**Enriched CSF3R mutations remove potential glycosylation sites.***A*, workflow for CSF3R library design and screening. *B*, substitutions at residue 618 from library 1 (NNN codon), grouped by hydrophobic (*red*), charged (*green*), uncharged (*blue*), and other (*yellow*) amino acids. *C*, possible codons at residue 618 with a single nucleotide mutation from the WT threonine (ACC, *blue*). *D*, the top 50 single nucleotide variants by frequency after screening library 2 (error-prone PCR). Axes use a log2 scale. Experiments were conducted at least two times with consistent results. CSF3R, colony-stimulating factor 3 receptor.

It is unknown why the T618I mutation is most prevalent in CNL. Two variables are the specific residue location and the biochemical properties of the substituted amino acids. Previous work by Zhang *et al.* suggested that hydrophobic residues, like isoleucine, at position 618 promote ligand-independent signaling (25). We therefore validated our high-throughput screen of CSF3R mutations by randomizing site 618 to allow any possible nucleotide combination at this position (termed an NNN codon). An advantage of this library design is that we can simultaneously test all 64 possible codons in one cell culture flask per condition. We cultured individual aliquots of the library under three conditions: (1) with mouse IL-3 to maintain the complete library as a starting point, (2) with human CSF3 to enrich for functional CSF3R variants that can signal in a ligand-dependent manner, and (3) with media without either ligand to enrich for activating mutations (Fig. 2*A*). Following sequencing of the cultured libraries, we then calculated the fold-change enrichment by dividing the frequency in the ligand-independent group by the frequency in either the IL-3 or CSF3 group (Table S1). As expected, hydrophobic and aromatic residues, most notably isoleucine, valine, phenylalanine, and tyrosine, are all enriched in the ligand-independent group (Fig. 2*B*). Alanine and methionine, which are often grouped with nonpolar residues but are less hydrophobic, were not enriched. Interestingly, amino acids with similar properties clustered together when plotted for ligand-independent enrichment over IL-3 and CSF3. For example, charged amino acids (aspartic acid, glutamine acid, lysine, arginine, and histidine) all demonstrate similar fold changes. Threonine and serine, the two amino acids which can be O-glycosylated, are the most depleted.

It is important to note that not all amino acid substitutions are possible with one nucleotide mutation at the codon for residue 618. Because we allowed for all possible codons, we could track codons individually for enrichment, including the nine possible single nucleotide variants from the WT ACC for T618. The clinically relevant T618I mutation results from a C to T transition (ACC to ATC). T618I may be prevalent in part because the C to T transition itself is more common in nature (26), but also because ATC is the only codon out of nine single-nucleotide mutations which encodes for a hydrophobic residue. The triplet ATC, which encodes isoleucine, is the only enriched codon in the ligand-independent group (Fig. 2*C*).

### Glycosite mutations are enriched across the 5FnIII domain

We next asked why T618I, and to a lesser extent T615A and N610H, are more common than mutations at other positions in the 5FnIII domain of CSF3R. We created an unbiased library of variants by randomly introducing mutations in the 5FnIII domain by error-prone PCR and again cultured cells with either IL-3, CSF3, or no ligand. While we expected to see enrichment of mutations at residue T618 due to its clinical relevance and its independent validation in oncogenesis, we strikingly observed that the most enriched sequences all removed a threonine, serine, or asparagine—the three amino acids with common glycosylation (Fig. 2*D*). We did not observe such an effect for other residues which could shape protein conformation. Interestingly, one of the most enriched variants—N579Y—had not previously been found in patients at the time of the screen but has since been identified in a rare case of CNL (27). We note that cells expressing WT CSF3R are found in the ligand-independent group but are depleted compared to the IL-3 and CSF3 groups and arise due to random mutations in the genome resulting from the transduction process.

### Glycoproteomics confirms the modification of amino acid residues

**Figure 3:**
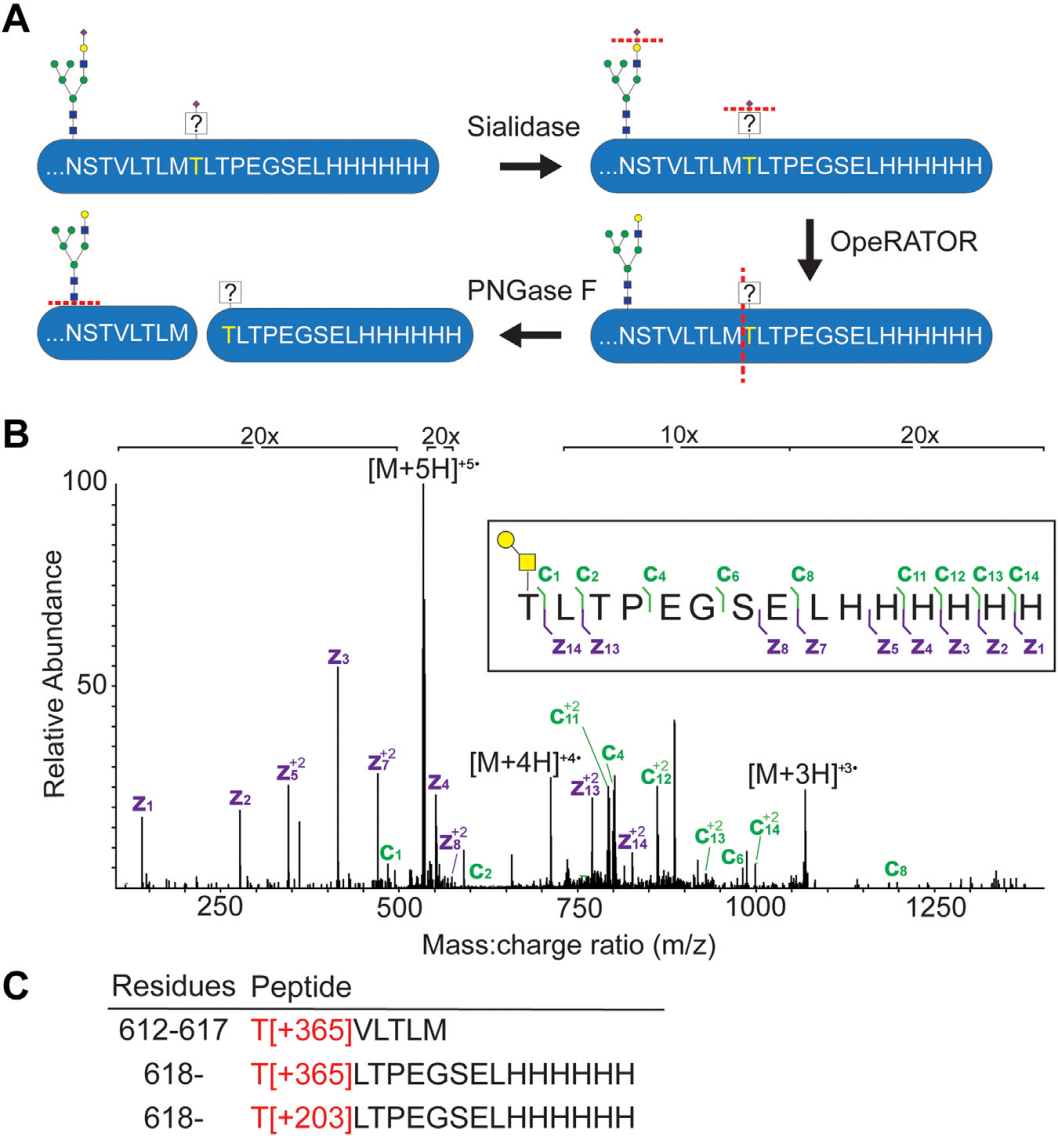
**WT threonine 618 is modified by glycosylation.***A*, workflow for digesting a soluble form of the WT CSF3R 5FnIII domain with sialidase, the protease OpeRATOR, and PNGase F. *B*, electron transfer dissociation (ETD) mass spectrum for the glycopeptide including the Gal-GalNAc (365 Da) glycan at T618. *C*, observed O-glycopeptides throughout the CSF3R 5FnIII sequence, including the GalNAc (203 Da) glycan at T618 and Gal-GalNAc glycan at T612. CSF3R, colony-stimulating factor 3 receptor; FnIII, fibronectin-III.

Given the long hypothesized model that T618I removes a glycosylation site along with the abundance of threonine, serine, and asparagine mutations in the error-prone library, we aimed to confirm glycosylation at these sites by MS. Short peptides have high charge density and facilitate site localization of glycans, but these peptides are challenging to create without standard protease (*i.e.* trypsin) cleavage sites near the residue of interest. We turned to the O-glycoprotease OgpA (commercially known as OpeRATOR) which cuts N terminally to threonine or serine residues if truncated O-linked glycosylation is present (28). Using a recombinant form of the WT CSF3R 5FnIII domain expressed in human Expi293F cells, we first treated samples with sialidase to remove sialic acid and enhance the activity of OgpA. Samples were then incubated with the OgpA protease as well as PNGase F to remove background glycosylation signal from N-linked glycans (Fig. 3*A*). We chose to employ a higher-energy collision dissociation product-dependent electron-transfer dissociation (HCD-pd-ETD) instrument method. Here, higher-energy collisional dissociation (HCD) was used to first identify signature glycan oxonium ions, which then triggers electron-transfer dissociation (ETD) fragmentation on the same precursor mass. Since ETD does not rely on collision for fragmentation, the glycan remains intact and allows for site-specific localization of the glycan. Using this instrument method and manual validation, we confirmed the presence of the disaccharide Gal-GalNAc glycan at threonine 618 (Fig. 3*B*). Other O-linked sugars, including the monosaccharide GalNAc at 618 and Gal-GalNAc at threonine 612, were identified across the CSF3R 5FnIII domain as well (Fig. 3*C*). Depending on the presence of sialic acid, these glycans could be the (sialyl-)Tn or T antigens, respectively. We analyzed N-linked glycosylation of CSF3R separately using parallel digests with trypsin and chymotrypsin proteases and an HCD-pd-EThcD MS/MS method (where EThcD is ETD followed by supplemental HCD activation) and verified the presence of glycans at residues N571, N579, and N610 (Fig. S1 and Table S2).

### Activating glycosite mutations are more common in CSF3R than related receptors

To determine how common such glycosite mutations are, we turned to proteins including the IL-6 family of receptors which have a similar structure to CSF3R. Of note, there is a high frequency of threonine and serine residues in the membrane-proximal FnIII domain of these receptors (Table 1). We selected proteins with an intracellular signaling domain and hydrophobic patches. We repeated the same library workflow to introduce random mutations into these domains and screen for ligand-independent BaF3 cell viability. For cells transduced with the single receptor libraries, only glycoprotein 130 (gp130) and leptin receptor (LEPR) cells expanded (Table S3). The outgrowth of gp130 cells was due to a large deletion (Δ260–621) (Fig. S2). This was independently observed by rationally removing the stalk region (29). LEPR cells expanded but did not grow in a validation experiment (Fig. S3). Therefore, growth was likely due to a passenger mutation within the gene sequence and a driver mutation elsewhere in the genome. As such, these variants are not relevant in the context of this work.

**Table 1:** Cytokine receptors with high frequencies of threonine and serine residues in the membrane-proximal domain

**Figure 4:**
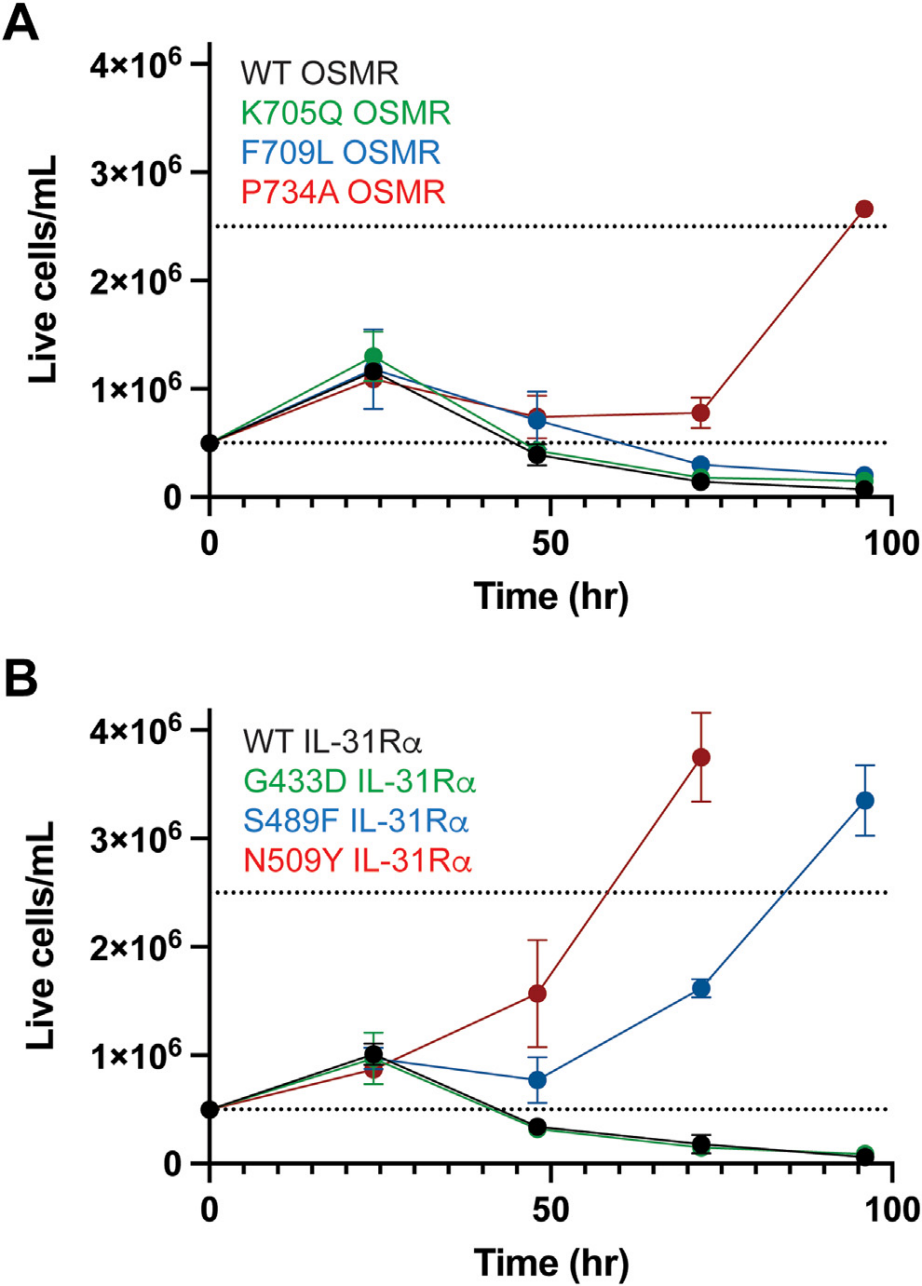
**Activating mutations in related receptors.** Outgrowth curves of BaF3 cells for OSMR variants transduced with WT IL-31Rα (*A*) and IL-31Rα variants transduced with WT OSMR (*B*). Values represent mean ± SD. Experiments were conducted at least two times with consistent results. IL, interleukin; IL-31Rα, interleukin-31 receptor alpha chain; OSMR, oncostatin-M receptor.

Many of these receptors typically form complexes with coreceptors for downstream signaling (30, 31), so we next repeated the screens by cotransducing the receptor library with its WT coreceptor: gp130/LIFR, LIFR/gp130, gp130/OSMR (oncostatin-M receptor), OSMR/gp130, OSMR/interleukin-31 receptor alpha chain (IL-31Rα), and IL-31Rα/OSMR. Using this approach, we identified enriched clones including OSMR P734A variants with and without additional mutations (K705Q and F709L), as well as IL-31Rα variants (T427N, G433D, S489F, and N509Y). We then validated these clones by transducing new BaF3 cells with the single mutations and growing them without ligand over time. Of the OSMR mutations, only P734A conferred ligand-independent growth in this outgrowth assay of BaF3 cells (Fig. 4*A*). The other variants, K705Q and F709L, were therefore passenger mutations or unable to contribute to cell proliferation without another mutation. While P734 is predicted to cap the transmembrane alpha helix and mutations to that site are likely to change activity (32), it is notable that many potential glycosites were not enriched in OSMR and that the corresponding proline mutation in CSF3R, P621, was not enriched. Of the IL-31Rα mutations, S489F and N509Y were validated by outgrowth assay (Fig. 4*B*). Although WT N509 is not part of a glycosylation sequon and cannot be modified by N-linked glycosylation, we have evidence that either T488 or S489 is modified by O-linked glycosylation (Fig. S4). The S489F mutation (numbered S521F by Lin *et al.*(33) due to a different isoform with 32 additional residues at the N-terminus, Fig. S5) is caused by a C to T transition like CSF3R T618I and has been reported in a case of primary cutaneous amyloidosis.

## Discussion

Studies of transmembrane receptors like CSF3R have often focused on ligand-binding domains, with only the three N-terminal domains represented in the published CSF3R crystal structure (34). The membrane-proximal region of cell surface receptors is a growing focus of research in structural biology, investigating the role of intermediate protein domains and stalk regions between ligand-binding sites and the transmembrane region (35). One example is the Tie2 receptor which, like CSF3R, was thought to dimerize in a ligand-dependent manner; however, an alternative ligand-independent mechanism was shown to be mediated by membrane-proximal FnIII domains (36).

We focused on CSF3R based on clinically relevant mutations in CNL and were particularly interested in the juxtamembrane domain given the results of the ligand-independent growth experiments (Fig. 1) and the high frequency of serine and threonine residues which creates the possibility for clustered O-linked glycosylation, as encountered in so-called mucin-like domains (37). Glycosylation has been shown to alter protein structure and function in multiple ways, including sterically occluding the underlying polypeptide as well as inducing specific polypeptide conformation (38, 39). Several groups have investigated the role of clustered O-glycans in either autolysis or cleavage by proteases, such as tumor necrosis factor-α by the metalloprotease ADAM17 (40, 41).

Our findings instead highlight the consequences of removing glycosylation from proteins near the membrane. Using unbiased screens, we demonstrated that enriched activating mutations in the 5FnIII domain of CSF3R predominantly replace a potential glycosite with a hydrophobic residue and that T618I is prevalent in part because of codon usage. Our findings indicate that membrane-proximal glycosylation serves as a “brake” to mediate receptor signaling. Rather than a binary “on/off” switch, the glycans allow for controlled signaling in the presence of ligand, and it is only when they are removed by mutation that aberrant signaling occurs. Of the seven receptors in this study, the phenomenon is most striking in CSF3R. One possible explanation is that CSF3R naturally forms a homodimer, rather than a heterodimer, in the presence of ligand. Consequently, any mutation could be found on both sides of the interface, which is especially important for the introduction of hydrophobic interactions. This is not the case for all homodimeric receptors, as we did not observe any mutations in LEPR that result in ligand-independent activity. For the receptors which form complexes with coreceptors, future studies could introduce mutations on both receptors at the same time, though such combinations would be unlikely in patients.

It is also important to note that the exact positioning of these glycosites is key, as residues at the interface will likely impact signaling more than those on the noninteracting surfaces. Affinity is often thought to be driven by a combination of select “hotspot” residues and additional, weaker interactions from other amino acids (42). Glycosylation may be similar, where sugar modification at key threonine, serine, or asparagine residues has an outsized effect on activity. When compared to IL-31Rα which also has an activating mutation at a potential glycosite, CSF3R may have a higher number of these glycosylation hotspots. As such, CSF3R may not necessarily be more prone to mutations in that domain, but when mutations arise, they could be more consequential. For this reason, full protein structures containing the membrane-proximal FnIII domains will provide additional insight into the mechanism of this activity.

In conclusion, this work confirms for the first time that there is O-linked glycosylation of CSF3R at WT T618. When mutated to isoleucine, we demonstrated that the 5FnIII domain–containing residue 618 is sufficient for ligand-independent activity. Isoleucine is one of the several hydrophobic residues which induce activation at this site but the only one that can be reached with a single nucleotide mutation to the WT threonine codon. Beyond residue 618, we identified activating mutations at other sites within the 5FnIII domain, all of which remove potential glycosylation sites. Though similar mutations can be found in additional receptors like IL-31Rα, they are more common in CSF3R, likely due to the positioning of these hotspot glycosites. The clinical relevance of threonine and serine mutations extends beyond CSF3R and CNL to other instances where dysregulated signaling leads to disease. For example, the S489F mutation in IL-31Rα leads to primary cutaneous amyloidosis. With growing glycoproteomics and genomic variation datasets, there is an opportunity to further explore such mutations in the future.

## Experimental procedures

### DNA cloning for transmembrane receptor constructs

CSF3R constructs were cloned into an MSCV-IRES-GFP (MIG) plasmid digested with XhoI (New England Biolabs R0146S). For the constructs with truncated domains, two inserts were amplified by PCR using Phusion polymerase (New England Biolabs M0530S) according to the manufacturer’s protocols: the CSF3R signal peptide (residues 1–24) and the C-terminal region including FnIII domains 1 to 5 (residues 125–836), domains 2 to 5 (residues 233–836), domains 3 to 5 (residues 334–836), domains 4 to 5 (residues 431–836), domain 5 (residues 530–836), or the control without an extracellular domain (residues 621–836). These fragments were gel extracted (Thermo Fisher Scientific K0692) according to the manufacturer’s protocols and added to Gibson assembly master mix (New England Biolabs E2611S). Stellar competent cells (Takara 636763) were transformed with the Gibson assembly products and spread on LB plates containing ampicillin. Individual colonies were miniprepped (Thermo Fisher Scientific K0503) and sequenced by Sanger sequencing (Elim Biopharm).

### Mammalian cell culture

HEK293T17 cells were cultured in Dulbecco's modified Eagle's medium (Thermo Fisher Scientific 11995073) supplemented with 10% fetal bovine serum (Thermo Fisher Scientific 26-140-079) and 1% penicillin-streptomycin (Thermo Fisher Scientific 15-140-122). BaF3 cells were cultured in RPMI 1640 (Thermo Fisher Scientific 11-875-119) supplemented with 10% fetal bovine serum, 1% penicillin-streptomycin, and 5 ng/ml recombinant murine IL-3 (PeproTech 213-13-10 μg). Expi293F cells (Thermo Fisher Scientific A14527) were cultured with shaking in Expi293 expression medium (Thermo Fisher Scientific A1435102). All cell lines tested negative for *mycoplasma* and were cultured at 37 °C in humidified incubators with 5% CO_2_ for HEK293T17 and BaF3 cells and 8% CO_2_ for Expi293F cells.

### Transfection and transduction

CSF3R constructs were used to transfect HEK293T17 cells. First, 135 μl of Opti-MEM I reduced serum medium (Thermo Fisher Scientific 31985062) was mixed with 10 μl of FuGENE 6 transfection reagent (Promega E2693). After 5 min, 2 μg of receptor plasmid (1 μg each for coreceptors) and 0.83 μg of the EcoPac packaging vector were added, and after 15 min, the complexes were dropped over a well of HEK293T17 cells in a 6-well plate. After two days, supernatant containing retrovirus for transductions was filtered with a 0.45 μm Steriflip (Thermo Fisher Scientific SE1M003M00).

For transduction, 1e6 BaF3 cells in 2 ml of complete media were mixed with 1 ml of media containing 10 ng/ml IL-3, 30 μl of 1 M Hepes (Thermo Fisher Scientific 15-630-080), 2 μl of polybrene (Millipore Sigma TR-1003-G), and 1 ml of filtered supernatant containing retrovirus. Cells were centrifuged at 2500 rpm for 90 min at 30 °C with no brake and cultured overnight at 37 °C. On the next day, 2 ml was removed from the well, centrifuged at 1100 rpm for 5 min, resuspended with 1 ml of media containing 10 ng/ml IL-3, and added back to the cells along with an additional 30 μl of 1 M Hepes, 2 μl of polybrene and 1 ml of fresh supernatant containing retrovirus. Centrifugation was repeated with the same settings, and cells were incubated overnight at 37 °C. On the following day, cells were centrifuged at 300*g* for 5 min and resuspended in complete media without retrovirus. After expansion, transduced, GFP-positive cells were isolated by fluorescence-activated cell sorting with an SH800 sorter (Sony), centrifuged at 300*g* for 5 min, resuspended in complete media, and cultured at 37 °C.

### Ligand-independent viability assay

Sorted BaF3 cells cultured in complete media containing IL-3 were washed three times by centrifuging at 1200 rpm for 5 min and resuspending in 5 ml of complete media without IL-3. After the washes, cells were diluted to a density of 6e4/ml, and 50 μl (3e3 cells) were added to 50 μl of media containing 2x of a serial dilution of human CSF3 (PeproTech 300-23) in a 96-well plate, for a total volume of 100 μl. After three days of incubation at 37 °C, 20 μl of CellTiter 96 AQueous One MTS reagent (Promega G3582) was added to cells. The absorbance at 490 nm was measured by a Synergy H4 plate reader (BioTek) according to the manufacturer’s protocols.

### Preparation of receptor libraries

For the randomized CSF3R library at residue 618, overlapping DNA oligos (IDT) were designed for the entire 5FnIII domain, with one containing the NNN codon at residue 618. The oligos were combined in a PCR assembly reaction (https://primerize.stanford.edu/protocol/#PCR). Ligations were used for cloning all libraries to maximize efficiency. Because SfiI, the restriction enzyme with a cut site closest to the 5FnIII domain, also cuts in the 3FnIII domain, the WT third and fourth FnIII domains were amplified by PCR. The 3-4FnIII fragments and randomized 5FnIII domain were digested with BsmBI-v2 (New England Biolabs R0739S), purified by PCR cleanup kit (Thermo Fisher Scientific FERK0702), combined with T4 ligase (Thermo Fisher Scientific 50-811-604), and then used as a template for PCR amplification of the complete insert. The full-length, WT CSF3R MIG plasmid and insert were digested with SfiI (New England Biolabs R0123S), ligated overnight with a 3:1 M ratio, and finally purified by PCR cleanup kit. Twenty-five microliters of Endura electrocompetent bacteria (Lucigen 60242-1) were transformed with 150 ng of library DNA using a 0.1 cm Gene Pulser electroporation cuvette (Bio-Rad 1652083) and the following settings: 10 μF, 600 Ω, 1800 V. The electroporated bacteria were then cultured in recovery medium (Lucigen 80026-1) for 1 h, then 50 ml of LB with ampicillin overnight. On the following day, DNA was purified by midiprep kit (Thermo Fisher Scientific K210014).

For the error-prone CSF3R library, mutations were randomly introduced across the 5FnIII domain (residues 530–627) by adding 2.5 μl each of 20 μM 8-oxo-dGTP TriLink BioTechnologies N-2034) and 20 μM dPTP (TriLink BioTechnologies N-2037) to a 50 μl Taq polymerase (Thermo Fisher Scientific 50-811-694) reaction. The product was gel extracted and then used as a template for further PCR amplification. This amplicon was digested and ligated as described above for transforming bacteria.

For the gp130, LIFR, OSMR, IL-31Rα, LEPR, and IL-2Rγ libraries, eBlocks (IDT) were designed with restriction enzyme cut sites near the FnIII region of interest. Constructs for WT receptors were cloned into MIG by Gibson assembly as described above with the BstBI (Thermo Fisher Scientific 50-812-170) and NotI (Thermo Fisher Scientific 50-811-2040) restriction sites. After sequence verification, plasmids were digested with BamHI-HF (New England Biolabs R3136S)/MfeI-HF (New England Biolabs R3589S) (gp130), MfeI-HF/AgeI-HF (New England Biolabs R3552S) (LIFR), AgeI-HF/PsiI-v2 (Thermo Fisher Scientific NC1704764) (OSMR), BsiWI-HF (Thermo Fisher Scientific NC1240162)/BsaBI (Thermo Fisher Scientific 50-812-207) (IL-31Rα), HpaI (New England Biolabs R0105S)/AgeI-HF (LEPR), or BamHI-HF/PsiI-v2 (IL-2Rγ), and the backbones were purified by gel extraction. The juxtamembrane FnIII domain of each receptor (gp130: 518-619, LIFR: 724-833, OSMR: 625-740, IL-31Rα: 421-519, LEPR: 740-839, IL-2Rγ: 156-262) was mutagenized by error-prone PCR as described above, amplified, and digested with the respective enzyme pair. All library constructs were then ligated and transduced into BaF3 cells as previously described.

### Screens of receptor libraries in BaF3 cells

BaF3 libraries were washed three times as described above to remove IL-3 from the media. After the washes, cells were resuspended in 4 ml at a density of 0.5e6/ml in complete media containing 5 ng/ml IL-3, 5 ng/ml CSF3, or no ligand and cultured at 37 °C. On the following day, cells were split back to 4 ml at 0.5e6/ml. Cells were counted every day and split to 4 ml at 0.25e6/ml after reaching a density greater than 1e6/ml. After cells were cultured for at least 1 week or until being passaged once, DNA was extracted from 2e6 cells using the QIAamp DNA Mini Kit (Qiagen 51304) according to the manufacturer’s protocols. The DNA encoding the FnIII domain of interest was amplified by PCR using primers with partial adapters for Amplicon-EZ (Azenta) analysis, purified by gel extraction, measured by a Qubit fluorometer (Thermo Fisher Scientific), and submitted to Azenta for next generation sequencing.

### Soluble protein expression and purification for MS

The sequence for the WT CSF3R 5FnIII domain (residues 530–626) with the CSF3R signal peptide (residues 1–24) and a C-terminal 6xHis tag was cloned into a pAdd2 mammalian expression vector at the EcoRI and XhoI sites by Gibson assembly, as described above. Expi293F cells were transfected with the pAdd2 5FnIII construct using the ExpiFectamine 293 kit (Thermo Fisher Scientific A14524) according to the manufacturer’s protocols. Six days after transfection, the supernatant was centrifuged at 300*g* for 5 min, adjusted to a pH of 8 with NaOH, centrifuged again at 3700*g* for 20 min, and filtered with a 0.22 μm bottle-top filter (EMD Millipore S2GPT02RE). His-tagged CSF3R protein was enriched by nickel-nitrilotriacetic acid agarose (Qiagen 30210) and further purified by size-exclusion chromatography on a Superdex 75 10/300 Gl column (Cytiva 17517401) with an AKTA Pure system (Cytiva).

### MS sample preparation

After purification, 7 μg of WT 5FnIII protein was incubated overnight at 37 °C in a final volume of 30 μl with 7 U of both SialEXO sialidase (Genovis G1-SM1-020) and OpeRATOR (Genovis G2-OP1-20) enzymes diluted in 20 mM Tris pH 7.5. After 18 h, the sample was diluted to 50 μl with ammonium bicarbonate buffer and incubated at 37 °C with 2 μl of PNGase F (New England Biolabs P0705S) diluted 1:100 in ammonium bicarbonate for an additional 7 h. The sample was combined with 69 μl of ammonium bicarbonate along with 1 μl of 1% ProteaseMAX surfactant (Promega V2072) for a total volume of 100 μl and incubated at 25 °C overnight. After 18 h, 0.3 μl of acetic acid was added to the sample before desalting with a C18 tip (Agilent A57203). The C18 tip was washed three times with 200 μl of methanol and then three times with 5% formic acid (FA). The sample was applied to the tip for 30 s, then collected and reloaded for a total of six times. The tip was washed three times with 200 μl of 5% FA and then eluted three times with 100 μl of 80% acetonitrile (ACN) and 5% FA. The sample was dried in a CentriVap Complete vacuum concentrator (Labconco) at 40 °C, resuspended in 5 μl of 0.1% FA, and frozen at −20 °C until analysis. For N-glycoproteomics, WT 5FnIII protein was resuspended in 100 mM Tris, and 2.5 μL of IMPa O-glycoprotease (New England Biolabs) was added for an incubation at 37 °C for 2 h, followed by the addition of tris(2-carboxyethyl)phosphine and chloroacetamide to a final concentration of 10 mM and 40 mM, respectively. One microgram of either trypsin (Promega) or chymotrypsin (Promega) was added and digests were incubated overnight at 37 °C. The next day, digested peptides were desalted using a 10 mg Strata-X cartridge (Phenomenex) by conditioning the cartridge with 1 ml ACN followed by 1 ml 0.2% FA in water. Peptides were acidified with FA and then loaded on to the cartridge, followed by a 1 ml wash with 0.2% FA in water. Peptides were eluted with 400 μL of 0.2% FA in 80% ACN, dried *via* lyophilization, and resuspended in 0.2% FA.

Because of low expression yields for the single IL-31Rα FnIII domain, the full extracellular domain of IL-31Rα (ACROBiosystems ILA-H52H7) was incubated in a similar fashion as described above. Twenty micrograms of the full IL-31Rα extracellular domain was resuspended in 100 mM Tris, and 2.5 μL of IMPa O-glycoprotease (New England Biolabs) was added for an incubation at 37 °C for 2 h, followed by addition of tris(2-carboxyethyl)phosphine and chloroacetamide to a final concentration of 10 mM and 40 mM, respectively. One microgram of chymotrypsin (Promega) was added and incubated overnight at 37 °C. The next day, digested peptides were desalted using the Strata-X cartridge protocol described above prior to drying *via* lyophilization. Desalted peptides were resuspended in 100 mM Tris, and 1 μl of a 1:20 dilution of PNGase F (New England Biolabs) was added for a 3-h incubation at 37 °C. Again, peptides were desalted, dried, and then resuspended in 0.2% FA for LC-MS/MS analysis.

### MS analysis

The frozen CSF3R sample was analyzed for O-glycopeptides as described by Shon *et al.*:

Samples were analyzed by online nanoflow liquid chromatography-tandem MS using an Orbitrap Fusion Tribrid mass spectrometer (Thermo Fisher Scientific) coupled to a Dionex Ultimate 3000 HPLC (Thermo Fisher Scientific). A portion of the sample (4.5 μl of 5 μl; 40%) was loaded *via* autosampler isocratically onto a C18 nano precolumn using 0.1% FA in water (“solvent A”). For preconcentration and desalting, the column was washed with 2% ACN and 0.1% FA in water (“loading pump solvent”). Subsequently, the C18 nano precolumn was switched in line with the C18 nano separation column (75-μm × 250-mm EASYSpray containing 2 μm C18 beads) for gradient elution. The column was held at 40 °C using a column heater in the EASY-Spray ionization source (Thermo Fisher Scientific). The samples were eluted at a constant flow rate of 0.3 μl/min using a 90-min gradient. The gradient profile was as follows (min:% solvent B, 2% FA in ACN): 0:3, 3:3, 93:35, 103:42, 104:95, 109:95, 110:3, 140:3. The instrument method used an MS1 resolution of 60,000 full width at half maximum at 400 m/z, an automatic gain control (AGC) target of 3e5, and a mass range from 300 to 1500 m/z. Dynamic exclusion was enabled with a repeat count of 3, repeat duration of 10 s, and exclusion duration of 10 s. Only charge states two to six were selected for fragmentation. MS2s were generated at top speed for 3 s. HCD was performed on all selected precursor masses with the following parameters: isolation window of 2 m/z, 30% collision energy, Orbitrap detection (resolution of 30,000), and an AGC target of 1e4 ions. ETD was performed if (1) the precursor mass was between 300 and 1000 m/z and (2) three of nine HexNAc or NeuAc fingerprint ions (126.055,138.055, 144.07, 168.065, 186.076, 204.086, 274.092, and 292.103) were present at ±0.1 m/z and greater than 5% relative intensity. ETD parameters were as follows: calibrated charge-dependent ETD times, 2e5 reagent target, and precursor AGC target 1e4.

Raw files were searched using Byonic by Protein Metrics against directed databases containing the recombinant protein of interest. Search parameters included semispecific cleavage specificity at the C-terminal site of R and K. Mass tolerance was set at 10 ppm for MS1s, 0.1 m/z for HCD MS2s, 0.35 m/z for ETD MS2s. Methionine oxidation (common 2), asparagine deamidation (common 2), and N-term acetylation (rare 1) were set as variable modifications with a total common max of 3 and a rare max of 1. O-glycans were also set as variable modifications (common 2), using the “O-glycan 6 most common” database. Cysteine carbamidomethylation was set as a fixed modification. Peptide hits were filtered using a 1% false discovery rate. All peptides were manually validated and/or sequenced using Xcalibur software (Thermo Fisher Scientific; https://www.thermofisher.com/order/catalog/product/OPTON-30965). HCD was used to confirm that the peptides were glycosylated, and ETD spectra were used for site-localization of glycosylation sites (43).

For analysis of N-glycopeptides from WT CSF3R 5FnIII and O-glycopeptides from IL-31Rα, HCD-pd-EThcD methods were used as previously described by Riley and Bertozzi:

Data were acquired using product-dependent triggering of EThcD scans (*i.e.*, an ETD with supplemental beam-type collisional activation) as previously described. Approximately 2 μg of peptides were injected on the column for each sample (one protein digest per run). Peptides were separated over a 25 cm Aurora Series UHPLC reversed phase LC emitter column (75 μm inner diameter packed with 1.6 μm, 160 Å, C18 particles, IonOpticks) that was heated to 40 °C by a Sonation PRSO-V2 column heater. A Dionex UltiMate 3000 RPLC nano system (Thermo Fisher Scientific) with an integrated loading pump was used for online liquid chromatography using mobile phases A (0.2% FA in water) and B (0.2% FA in ACN). Peptides were loaded onto a trap column (Acclaim PepMap 100 C18, 5 μm particles, 20 mm length, Thermo Fisher Scientific) at 5 μl/min, which was put in line with the analytical column 5.5 min into the acquisition. Gradient elution was performed at 300 nl min^−1^. The gradient was held at 0% B for the first 6 min of the analysis, followed by an increase from 0% to 5% B from 6 to 6.5 min, an increase from 5% to 22% B from 6.5 to 156.5 min, an increase from 22% to 90% B from 156.5 to 160 min, isocratic flow at 90% B from 160 to 164 min, and a re-equilibration at 0% for 16 min for a total analysis time of 180 min. Eluted peptides were analyzed on an Orbitrap Fusion Tribrid MS system (Thermo Fisher Scientific). Precursors were ionized using a nanospray flex ionization source (Thermo Fisher Scientific) held at +2.2 kV compared to ground, and the inlet capillary temperature was held at 275 °C. Survey scans of peptide precursors were collected in the Orbitrap from m/z 400 to 1800 with a normalized AGC target of 100% (400,000 charges), a maximum injection time of 50 ms, and a resolution of 60,000 at m/z 200. Monoisotopic precursor selection was enabled for peptide isotopic distributions, precursors of z = 2 to 8 were selected for data-dependent MS/MS scans for 3 s of cycle time, and dynamic exclusion was enabled with a repeat count of 2, repeat duration of 20 s, and exclusion duration of 20 s. Priority filters were set to favor highest precursor charge states and lowest precursor m/z values. An isolation window of 2 m/z was used to select precursor ions with the quadrupole. EThcD scans were collected in product-dependent fashion, where the presence of oxonium ions (m/z 126.055, 138.0549, 144.0655, 168.0654, 186.076, 204.0865, 274.0921, 292.1027, and 366.1395) in a “scouting” HCD MS/MS scan triggered acquisition of a second MS/MS scan. The “scout HCD” scan used an automated scan range determination and a first mass of 100 Th, a normalized collision energy of 36, a normalized AGC target value of 100% (50,000 charges), a maximum injection time setting of Auto (54 ms), and a resolution of 30,000 at m/z 200. If at least four of the nine listed oxonium ions were present in the scout HCD scan within a ±15 ppm tolerance and were among the 20 most intense peaks, an EThcD MS/MS scan was triggered that used calibrated charge-dependent parameters for calculating reagent AGC targets and ion–ion reaction times, a supplemental collision energy of 25, a scan range of m/z 200 to 4000, a maximum injection time of 400 ms, a normalized AGC target of 200% (100,000 charges), and a resolution of 60,000 at m/z 200 (44).

N-glycoproteomic files from WT 5FnIII were searched using Byonic by Protein Metrics against directed databases containing CSF3R. Search parameters included fully specific cleavage specificity C-terminal to F, Y, W, and L residues and a mass tolerance of 10 ppm for precursor and 20 ppm for product ions. Variable modifications were methionine oxidation (rare 2) with a total rare max of 2 and a library of 183 N-glycans (common 1) with a total common max of 2. A fixed modification was cysteine carbamidomethylation. Hits were filtered and manually validated with Xcalibur as previously described. O-glycoproteomic files from IL-31Rα were processed using O-Pair Search (45) with directed databases containing IL-31Rα. Settings were the “Glyco Search” selection with an O-glycan database of 22 glycans and the “Maximum OGlycan Allowed” at 4. Variable modifications were methionine oxidation and asparagine deamidation. Additional parameters were mass tolerances of 10 ppm for precursor and 20 ppm for product ions, with a minimum score allowed of three. A protease setting of IMPa-chymotrypsin was created with N-terminal cleavage to S and T residues and C-terminal cleavage to F, Y, W, and L residues with fully specific parameters. Filters included only target matches (T) and a q-value <0.01, and sequences of interest were then manually inspected and validated.

### Outgrowth validation assay

BaF3 cells were transduced with receptor constructs for single mutations, as described above. After washing cells three times to remove IL-3, cells were resuspended in 4 ml of complete RPMI media without ligand at a density of 0.5e6/ml. Cells were counted every day until expanding five-fold to 2.5e6/ml. Extracted DNA was then reanalyzed by Sanger sequencing to confirm that no new mutations had been acquired.

## Data availability

The mass spectrometry proteomics data for Figure 3 have been deposited to the ProteomeXchange Consortium *via* the PRIDE partner repository (46) with the dataset identifier PXD035664.

## Supporting information

This article contains supporting information (33).

## Conflict of interest

C. R. B. is a cofounder and scientific advisory board member of Lycia Therapeutics, Palleon Pharmaceuticals, Enable Biosciences, Redwood Biosciences (a subsidiary of Catalent), OliLux Bio, Grace Science LLC, and InterVenn Biosciences. J. R. C. is a cofounder and equity holder of Combangio, Inc. (now Kala Pharmaceuticals), xCella Biosciences (now OmniAb), and Red Tree Venture Capital; has financial interests in Aravive, Inc.; is a member of the Board of Directors of OmniAb and Revel Pharmaceuticals; and is a Board Observer at Excellergy Therapeutics, Tachyon Therapeutics, and Acrigen Biosciences. J. E. M. receives funding from Gilead Sciences, Kura Oncology, Blueprint Medicines and is involved in a collaboration with Ionis Pharmaceuticals. S. A. M. is a consultant for InterVenn Biosciences. All other authors declare no conflicts of interest with the contents of this article.

## Supporting information

Supporting Table S3 and Figures S1–S5

Supporting Table S1 Sequencing data for Figure 2.

Supporting Table S2 N-glycopeptides at N571, N579, and N610 of wild-type CSF3R.
