# Supplementary material for: Mutational screens highlight glycosylation as a modulator of colony-stimulating factor 3 receptor (CSF3R) activity
Source: J Biol Chem. 2023 Apr 26;299(6):104755. doi: 10.1016/j.jbc.2023.104755 (PMC10245049; doi:10.1016/j.jbc.2023.104755)
Supplement: Supporting Table S3 and Figures S1–S5 [file mmc1.docx]

**Supporting Information**


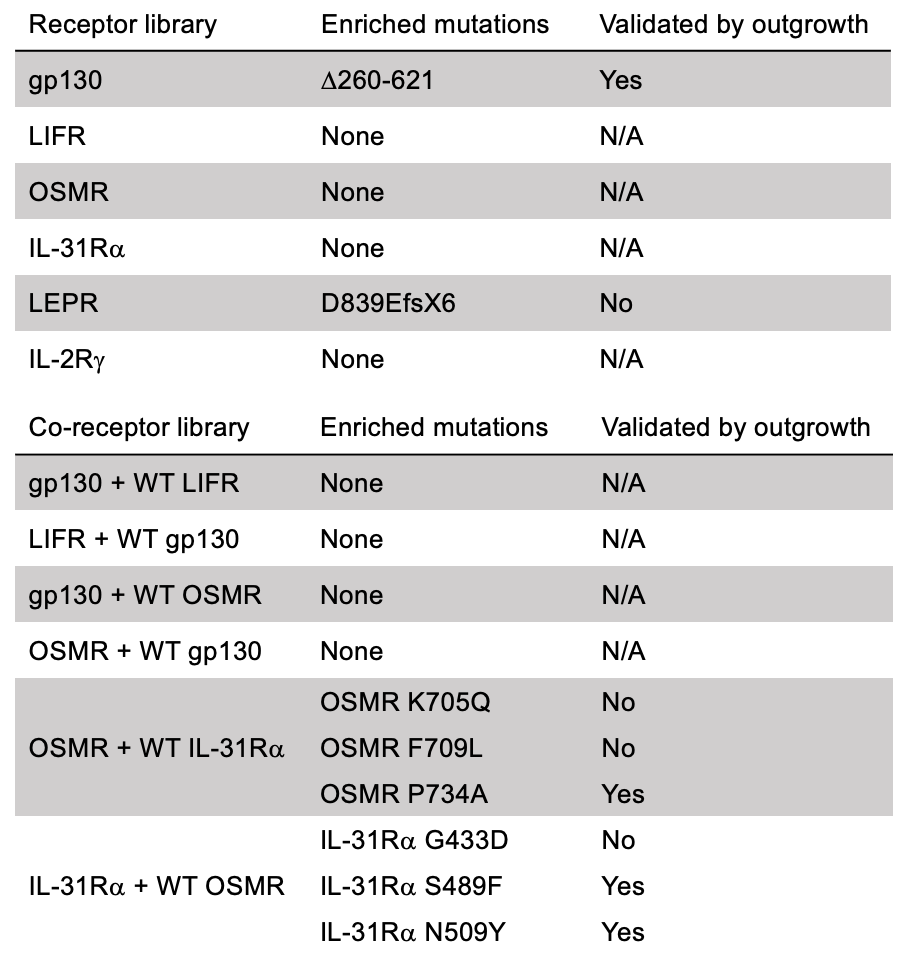


**Supporting Information Table S3** – Summary of mutations from (co)receptor libraries.


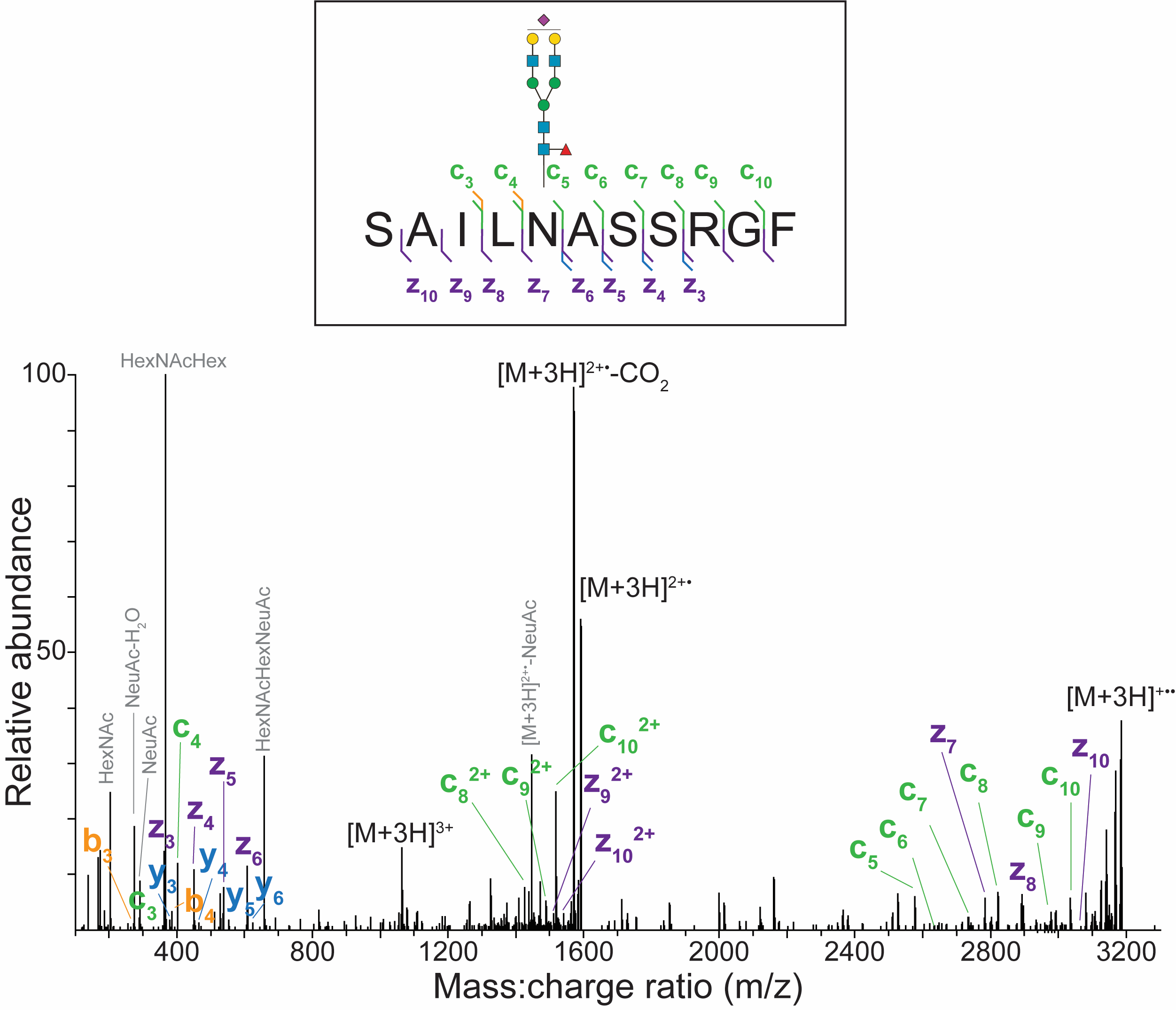


**Supporting Information Figure S1** –Example spectrum for a complex N-glycan with fucosylation and sialylation at CSF3R N579.


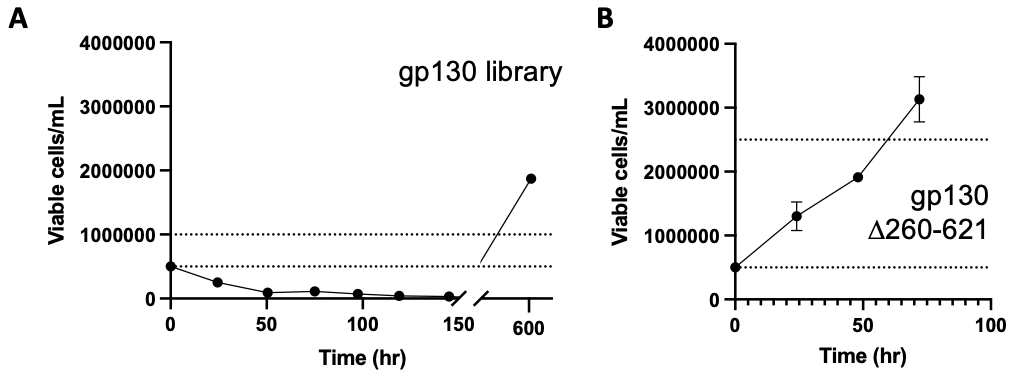


**Supporting Information Figure S2** – Screen of gp130 library (A) and validation on enriched gp130 ∆260-621 clone by outgrowth assay (B).


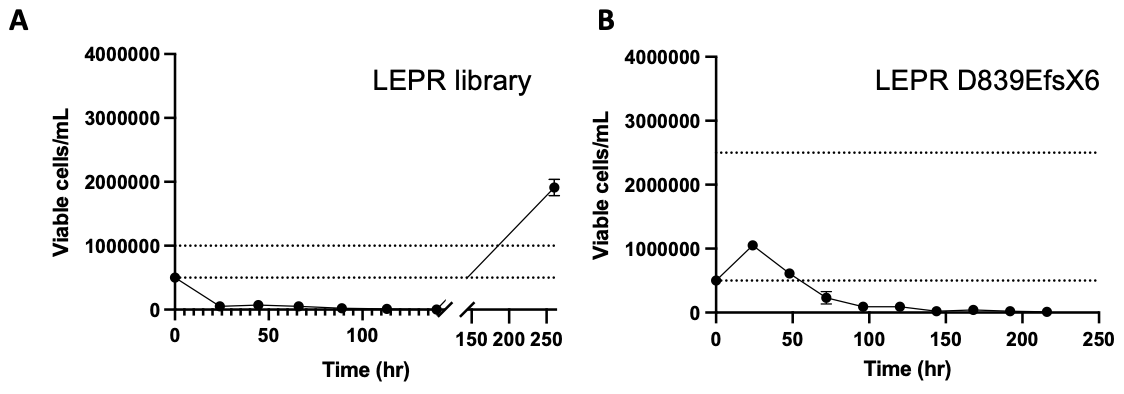


**Supporting Information Figure S3** – Screen of LEPR library (A) and outgrowth test for enriched frameshift mutation, LEPR D839EfsX6 (B).


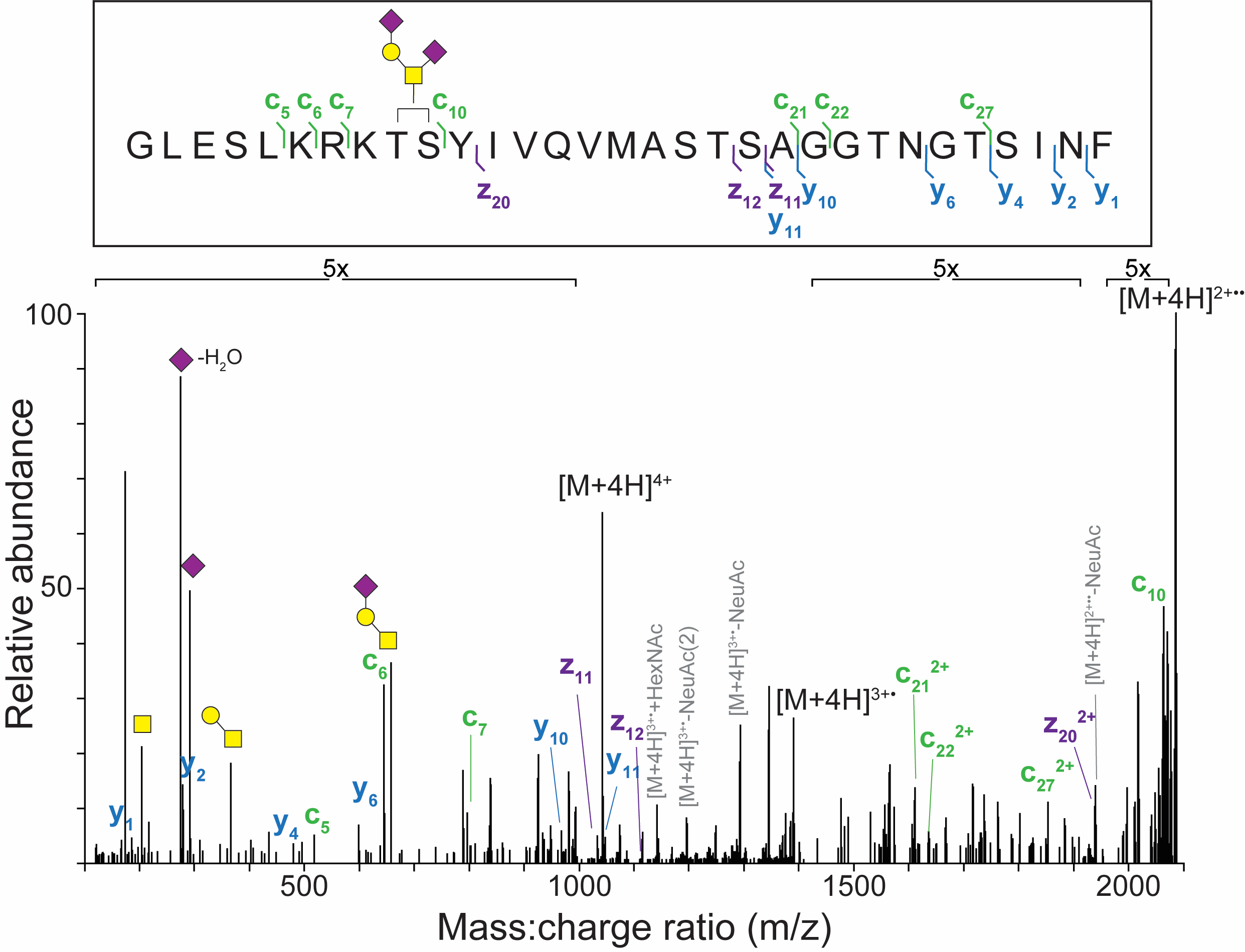


**Supporting Information Figure S4** – O-glycopeptide spectrum with evidence for the disialyl-T antigen at T488 or S489 of IL-31Rα.


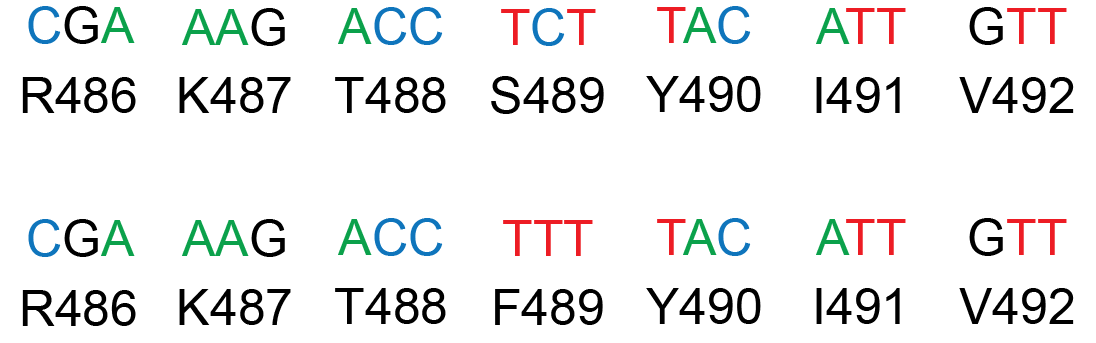


**Supporting Information Figure S5** – Alignment of canonical IL-31Rα sequence to the region featured in Figure 3 by Lin et al.^33^ The NM_139017 isoform includes 32 additional residues (MCIRQLKFFTTACVCECPQNILSPQPSCVNLG) at the N-terminus. Consequently, S489 corresponds to S521.
